# Calcium, mitochondria, and the pathogenesis of ALS: the good, the bad, and the ugly

**DOI:** 10.3389/fncel.2013.00199

**Published:** 2013-10-31

**Authors:** Manoj Kumar Jaiswal

**Affiliations:** Department of Anatomy, Physiology, and Genetics, School of Medicine, USUHSBethesda, MD, USA

**Keywords:** amyotrophic lateral sclerosis, calcium homeostasis dysregulation, mitochondrial dysfunction, neurodegeneration, calcium buffer, selective vulnerability, motor neuron disease

Amyotrophic lateral sclerosis (ALS) is a devastating neurodegenerative disease characterized by the selective and progressive loss of upper and lower motor neurons (MNs) of the brainstem, spinal cord and cerebral cortex. Key discoveries in the field of ALS have exponentially increased since it was first described in 1869 by Jean Martin Charcot, and the identification in 1993 of the first ALS-causing mutations in the gene encoding the well-studied antioxidant enzyme Cu, Zn superoxide dismutase (SOD1). The etiology of sporadic ALS largely remains unknown and the mechanisms of MN degeneration are still being investigated. The only FDA drug approved for the treatment of ALS, riluzole only modestly benefits patients, but multiple drugs are currently in the development pipeline and in human ALS clinical trials. It has become obvious that the lack of understanding of the precise mechanisms of motor neuron degeneration presents a major obstacle in the development of effective therapies for ALS. Altered calcium homeostasis and calcium signaling pathway activation is one potential mechanism that accounts for at least three major and interrelated toxic pathways: oxidative stress, mitochondrial dysfunction and neuroinflammation in neurodegenerative diseases such as ALS.

The aim of this special topic was to determine the role of cellular Ca^2+^ homeostasis and mitochondrial signaling pathways in selectively vulnerable MNs in ALS. In fact, recent evidence suggests that abnormalities in cellular Ca^2+^ signaling are common features in the pathogenesis of a range of neurodegenerative disorders, including ALS. It is well known that Ca^2+^ is one of the most relevant intracellular messengers, being essential in neuronal development, synaptic transmission and plasticity, as well as in the regulation of various metabolic pathways in the brain level. In subtypes of ALS associated with the SOD1 mutation and the sporadic disease, there have been several reports indicating that the involvement of mitochondria in pathogenesis includes the generation of intracellular free radical species, ultrastructral changes in mitochondrial morphology, swollen and vacuolar mitochondria and increased activity of complex I, III and IV in the Upper and lower MNS, frontal cortex and spinal cord (von Lewinski and Keller, 2005). Impaired spinal cord and vulnerable individual spinal MNs have also been reported. It has been proposed that this damage triggers the functional decline of MNs and the onset of pathology in ALS. The loss of mitochondrial membrane potential, excitotoxic stimulation of AMPA/kainite receptors and age related MN injury reported by many groups may contribute to ALS pathogenesis.

Further evidence for the involvement of disruption of intracellular Ca^2+^ homeostasis arises from several studies of cellular and experimental animal models of ALS in which Ca^2+^ binding proteins such as calbindin-D28K and parvalbumin in MN populations are lost early (hypoglossal, spinal, and lower cranial MNs). These findings are in good agreement with a quantitative comparison of Ca^2+^ homeostasis where low cytosolic Ca^2+^ buffering capacity acts as an important risk factor for degeneration and in contrast an increase in cytosolic Ca^2+^ buffering capacity could protect vulnerable MNs from degeneration both *in-vitro* and *in-vivo*. Several lines of evidence support altered Ca^2+^ homeostasis leading to MN degeneration in ALS, notably disturbance of glutamate neurotransmission and subsequent glutamate triggered Ca^2+^ entry, increased extracellular glutamate levels probably due to reduced glial glutamate uptake caused by oxidative damage to EAAT2, study of fALS in cell lines and animal mouse models where potential mechanism for Ca^2+^disruption is inhibition of glial glutamate transport by mSOD1 similar to those proposed for sALS. Evidence also suggests that ROS generated in MNs can cross the plasma membrane and cause oxidative disruption of glutamate transporters in neighbouring astrocytes. However, in ALS pathology, the nature of dysfunction is highly controversial after recent findings where non-cell autonomous effect of glia on MNs in an embryonic stem cell-based ALS model and astrocytes expressing ALS-linked mSOD1 that release factors selectively toxic to MNs was shown. The data currently available so far leave several important questions unanswered; these include: (1) does the presence of mSOD1 cause morphological abnormalities of mitochondria when expressed at physiological levels? (2) Does the mitochondrial Ca^2+^ sequestration source specificity and spatiotemporal properties of [Ca^2+^]i signaling varies at physiological levels? (3) What are the functional consequences of any changes in mitochondrial function on Ca^2+^ homeostasis in the presence of mutant SOD1 gene?

Combining the lessons from multiple animal models as well as cell culture model used to determine pathogenic mechanisms in ALS, the central insight is that selective vulnerability of MNs likely arises from a combination of several mechanisms; two of them, mitochondrial dysfunction and Ca^2+^ homeostasis are prominent (Jaiswal and Keller, 2009; Jaiswal et al., 2009). Documenting the involvement and importance of mitochondrial dysfunction and Ca^2+^ homeostasis we believe that MN possess large number of voltage and ligand gated Ca^2+^ channels that, when activated, cause rapid Ca^2+^ influx, which, in part because of relatively weak cytosolic Ca^2+^ buffering, results in mitochondrial Ca^2+^ overload and strong ROS generation. Alternatively, the extrusion mechanisms, characterized by the plasma membrane calcium ATPase, Na^+^/Ca^2+^ exchanger and accumulation by intracellular organelles contribute to the removal of calcium ions. Furthermore, their selective loss goes a long way explaining the oxidative damage, mitochondrial abnormalities and apoptotic contributions observed in ALS MNs. Chronic mitochondrial membrane depolarisation due to Ca^2+^ entry can cause the release of pro-apoptotic proteins and activate enzymes involved in apoptotic pathways.

In the last few years, attention was drawn to the role of mitochondria as an efficient regulator of cytosolic calcium signals. Further studies with mitochondria targeted calcium probes indicate a rapid, dramatic increase in free intramitochondrial calcium. This uptake by mitochondria has an immense effect on the metabolic state of the cell as it can up regulate the activity of the enzymes in oxidative metabolism. The malformations in mitochondrial structure and massive vacuoles derived from degenerating mitochondria found in post mortem human ALS samples further strengthen the proposition. Although the exact molecular mechanism is still not known, we hypothesize that vulnerability to ALS is a consequence of specific physiological features, particularly highly specialized Ca^2+^ homeostasis, continuous activity-dependent Ca^2+^cycling and the predominant role of mitochondria in buffering Ca^2+^ transients. Definitive evidence for this will have to wait for further studies. Nevertheless, if Ca^2+^ homeostasis and mitochondrial defects were found to play a role in disease pathogenesis, this would have broad ranging implications for therapy. Further studies will therefore be of considerable interest in attempting to understand the pathogenesis of cell death in ALS.
